# Evaluation of Skinfold Techniques in People with Down Syndrome: Development of a New Equation

**DOI:** 10.3390/ijerph20105831

**Published:** 2023-05-16

**Authors:** Brett S. Nickerson, Michael R. Esco, George Schaefer

**Affiliations:** 1 School of Health and Rehabilitation Sciences, The Ohio State University, Columbus, OH 43210, USA; 2 Department of Kinesiology, The University of Alabama, Tuscaloosa, AL 35487, USA; mresco@ua.edu; 3 Department of Kinesiology, Auburn University at Montgomery, Montgomery, AL 36117, USA

**Keywords:** trisomy 21, body composition, adiposity, anthropometric

## Abstract

The primary aim of this study was to evaluate the accuracy of skinfold thickness (SFT) measurements for the estimation of %Fat when compared to dual energy X-ray absorptiometry (DXA) in individuals with Down syndrome (DS). The secondary aim was to develop a new SFT-based body fat equation (SFT_NICKERSON_). SFT-based %Fat was estimated using a body fat equation from González-Agüero (SFT_G-A_) and body density conversion formulas from Siri (SFT_SIRI_) and Brozek (SFT_BROZEK_). Criterion %Fat was measured via DXA. SFT_G-A_, SFT_SIRI_, and SFT_BROZEK_ were significantly lower than DXA (mean differences ranged from −7.59 to −13.51%; all *p* < 0.001). The SEE values ranged from 3.47% (SFT_BROZEK_) to 8.60% (SFT_G-A_). The 95% limits of agreement were greater than ±10% for all comparisons. Mid-axilla and suprailium were significant predictors of %Fat (both *p* < 0.05). %Fat SFT_NICKERSON_ = 10.323 + (0.661 × mid-axilla) + (0.712 × suprailium). Age and all other skinfold sites were not statically significant in the regression model (all *p* > 0.05). Current findings indicate that SFT_G-A_, SFT_SIRI_, and SFT_BROZEK_ erroneously place an individual with excessive adiposity in a normal healthy range. Accordingly, the current study developed a new equation (SFT_NICKERSON_) that can easily be administered in people with DS in a quick and efficient time frame. However, further research is warranted in this area.

## 1. Introduction

Accurately assessing the adiposity of individuals with Down syndrome (DS) should be a top priority of health professionals due to the unfavorable body composition profiles observed in this special population. For instance, adults with DS are twice as likely to be obese when compared to adults without DS [1]. The health consequences associated with excessive fat accumulation include cardiovascular diseases, type II diabetes, metabolic syndrome, etc. [2,3]. Consequently, the high prevalence of obesity among individuals with DS underscores the importance of selecting a body composition method that is accurate for adiposity measurements (%Fat). Specifically, an accurate method may prove useful for early detection of obesity, which can subsequently be useful in developing lifestyle interventions designed to avoid chronic diseases that occur because of excessive adiposity accumulation.

Imaging-based methods such as magnetic resonance imaging, computed tomography, and dual energy X-ray absorptiometry (DXA) are often utilized in body composition research as criterion measures [4,5,6,7]. One benefit of these methods is the ability to assess total and regional body composition. For instance, the ability to carry out appendicular and trunk analysis of body composition is likely a reason why these clinical methods are highly desired and utilized in validation research and clinical settings [8,9]. Unfortunately, the assessment of body composition in special populations, such as individuals with DS, can be problematic with imaging-based methods. For example, children with DS visit doctors more often than typically developing children. Accordingly, the aforementioned imaging devices could remind people with DS of those medical visits, which may induce a feeling of discomfort or anxiety commonly experienced when visiting doctors’ office. As a result, alternative approaches that can be easily administered and avoid inducing a stressful environment are more ideal.

Skinfold thickness (SFT) is a field approach that may be able to overcome the stressful environment often induced when administering imaging-based assessments of body composition. Nonetheless, there are various concerns that could influence the precision of methods such as SFT, especially when being administered in a DS population. For instance, one issue that may influence the accuracy of existing methods is the fact that body composition differs between individuals with and without DS [10,11]. This could be problematic since many methods that have been used to estimate %Fat, including SFT, have been developed in the general population [12,13,14,15,16,17,18]. These findings suggest that external validation in special populations, including individuals with DS, is necessary before widespread use.

The estimation of %Fat from SFT measurements is determined via various anatomical sites [19,20,21,22,23,24]. For example, three-, four-, and seven-site measurements can be used for the estimation of %Fat. Methods such as SFT can use body density (D_b_) conversion formulas to predict adiposity or regression equations [23,24,25,26]. The advantage of SFT over more sophisticated clinical-based methods, such as DXA, is the ease of administration and affordability. Although thoroughly evaluated in various populations and races/ethnicities, the evaluation of SFT has been extremely limited in people with DS.

Prior research revealed that existing SFT-based equations may not be accurate for the estimation of %Fat in children with DS [27]. Thus, more recent research sought to develop a new SFT equation (SFT_G-A_) specifically for children with DS [28]. Nonetheless, many questions need to be answered prior to using SFT_G-A_ in individuals with DS. For instance, it is unknown whether SFT_G-A_ is accurate for adults with DS. In addition, it is unknown whether SFT_G-A_ performs better than commonly used D_b_ conversion formulas such as Siri (SFT_SIRI_) [26] and Brozek (SFT_BROZEK_) [25]. Lastly, it is worth noting that SFT_G-A_ only includes a triceps measurement whereas most SFT equations include three- to seven-site measurements, as previously noted. Altogether, a thorough evaluation of SFT_G-A_, SFT_SIRI_, and SFT_BROZEK_ needs to be conducted in individuals with DS to identify if either of these methods are accurate or whether a new equation should be developed. Therefore, the primary aim of this study was to evaluate the accuracy of SFT measurements for the estimation of %Fat when compared to DXA in individuals with DS. The secondary aim was to develop a new SFT-based body fat equation (SFT_NICKERSON_). Due to the differences in body composition between individuals with and without DS [29], we hypothesized SFT_SIRI_ and SFT_BROZEZ_ would not be accurate for our study sample. In addition, the use of only one skinfold site (i.e., triceps), led to the hypothesis that the DS specific equation from SFT_G-A_ would not be accurate when applied to a group of males and females with DS.

## 2. Materials and Methods

### 2.1. Participants

In total, 20 individuals with DS (males: *n* = 9; females: *n* = 11) had body composition estimated via DXA and SFT. Subjects < 18 years of age were classified as children (*n* = 6). The race/ethnicity of the study sample consisted of non-Hispanic whites (*n* = 17) and non-Hispanic blacks (*n* = 3). Participants characteristics are depicted in Table 1. Recruitment for the study included researchers visiting local DS community centers to discuss the project with parents/guardians with children that might be interested in volunteering. Further, recruitment occurred via word of mouth and flyers. The criteria for participation included being at least 10 years of age and diagnosed with DS. All participants and guardians provided written consent. Institutional review board approval for subject participation was approved by the host university.

### 2.2. Procedures

Height was measured (to the nearest 0.1 cm) with a wall-mounted stadiometer (SECA; Seca Instruments Ltd., Hamburg, Germany). Body mass was measured to the nearest 0.10 kg with a digital scale (Tanita BWB-800A, Tanita Corp., Tokyo, Japan). SFT-based %Fat was estimated using SFT_G-A_ [28], SFT_SIRI_ [26], and SFT_BROZEK_ [25] whereas criterion %Fat was measured via DXA.

### 2.3. Skinfolds

All skinfold measurements were conducted on the right side of the body with a calibrated Lange caliper. Seven-site measures were taken at the chest, mid-axilla, triceps, abdomen, suprailium, subscapular, and thigh in order to determine D_b_ [30]. Next, D_b_ was converted to %Fat using Siri’s [26] and Brozek et al.’s [25] conversion formulas. Lastly, the triceps skinfold measurement was used to predict %Fat for SFT_G-A_ [28].

%Fat SFT_G-A_ = (0.97 × triceps) − (0.869 × sex) + 15.6 %Fat SFT_BROZEK_ = (4.57/D_b_) × 100 %Fat SFT_SIRI_ = (4.95/D_B_) × 100 Sex: 0 = Female; 1 = Male

### 2.4. Dual Energy X-ray Absorptiometry

Participants also had their %Fat measured with DXA (GE Lunar Prodigy, Madison, WI, USA). The DXA machine was calibrated according to the manufacturer’s instructions via a standard calibration block prior to each scan. The DXA quality control procedures were followed according to manufacturer guidelines. All subjects reported to the laboratory and were asked to remove any metal objects (e.g., jewelry, coins, etc.). Volunteers wore light cotton clothing free from metal during the scan and were instructed to lie supine on the scanning bed with hands by their sides in a neutral position. During body scans, subjects were asked to remain motionless, while Velcro straps were situated around the ankles and knees. Scans lasted approximately 6 to 10 min. The same researcher positioned all participants on the DXA scanning bed. In addition, the trained researcher analyzed each scan to adjust software-determined regions of interest prior to producing the body composition reports.

### 2.5. Statistical Analyses

All statistical analyses were performed using IBM SPSS Statistics v. 28.0 (IBM Corp., Armonk, NY, USA), and data visualizations were created using Microsoft Excel (Microsoft Corp., Redmond, WA, USA). The magnitude of the mean differences was assessed using Cohen’s *d* statistics [31] and evaluated with Hopkins’ effect size scale [32]. The scale for effect sizes was 0 to <0.2 for trivial, 0.2 to <0.6 for small, 0.6 to <1.2 for moderate, 1.2 to <2.0 for large, and ≥2.0 for very large. The agreement between SFT and DXA was assessed using Pearson’s correlation (*r*), standard error of the estimate (SEE), and constant error (CE). Additionally, Bland–Altman analyses [33] and linear regression modeling (enter method) were used to identify the 95% limits of agreement (LOA) and proportional bias for %Fat. Proportional biases were determined by the estimated slope of the regression line. A slope of 0 would indicate no proportional bias. The strength of association between outcome variables (*r*), as estimated by DXA and SFT, used a scale of 0 to 0.19 as very weak-to-no association, 0.20 to 0.39 as weak, 0.40 to 0.59 as moderate, 0.60 to 0.79 as strong, and 0.80 to 1.00 as very strong-to-perfect [32,33]. Alpha levels were set a priori at a value of <0.05. Values for all outcome variables are displayed as mean ± standard deviation.

Pearson’s correlations were also used for establishing the relationship between DXA-derived %Fat and the sum of skinfolds (7-site measurements). Lastly, stepwise regression analysis was performed to obtain a prediction model whereby DXA-derived %Fat was used as the dependent variable, and the 7 skinfold sites, sex, and age were used as prediction variables. The criterion for inclusion (addition and retention) of a predictor variable was significant at the *p* = 0.05 level.

## 3. Results

The agreement of SFT_G-A_, SFT_SIRI_, and SFT_BROZEK_, when compared to DXA is displayed in Table 2. The mean values for all SFT measurements were significantly lower than DXA (CEs ranged from −7.59 to −13.51%; all *p* < 0.001). The correlation coefficients were strong (SFT_A-G_) and very strong-to-perfect (SFT_SIRI_ and SFT_BROZEK_). The SEE values ranged from 3.47% (SFT_BROZEK_) to 8.60% (SFT_G-A_). Further, the 95% LOAs were greater than ±10% for all comparisons. Lastly, there was significant proportional bias observed when estimating %Fat via SFT_SIRI_ (*r* = −0.58) and SFT_BROZEK_ (*r* = −0.69).

The correlation coefficients between DXA-derived %Fat and each of the SFT measurement site are displayed in Table 3. All correlations were statistically significant and were smallest for chest (*r* = 0.518; *p* = 0.19) and largest for mid-axilla (*r* = 0.811, *p* < 0.001). In the first step of the regression, sum of mid-axilla explained 66% of the variance in DXA-derived %Fat. In the subsequent step, suprailium increased the explained variance to 83% (Table 4). Age and all other skinfold sites were not statically significant for either step of the regression analysis (all *p* > 0.05). Thus, the final model revealed two predictor variables of %Fat as follows:

%Fat SFT_NICKERSON_ = 10.323 + (0.661 × mid-axilla) + (0.712 × suprailium)

## 4. Discussion

The primary aim of this study was to evaluate the accuracy of SFT measurements for the estimation of %Fat when compared to DXA in individuals with DS. The secondary aim was to develop a new SFT-based body fat equation. The current study revealed an alarming amount of error when estimating %Fat via SFT. Despite a population-specific equation, SFT_G-A_ underestimated %Fat by a staggering amount in the group of individuals with DS (CE = −7.59%). Further, SFT_G-A_ was found to underestimate %Fat by more than 20% when evaluated at the individual level. The group error (CE) was even more profound when evaluating SFT_SIRI_ (CE = −12.90%) and SFT_BROZEK_ (CE = −13.51%). These findings suggest that %Fat is grossly underestimated by SFT_SIRI_ and SFT_BROZEK_ as well. In addition, SFT_SIRI_ and SFT_BROZEK_ yielded significant proportional bias which suggests %Fat is underestimated to an even greater extent at higher DXA-based %Fat values. Altogether, these findings support our initial hypotheses that D_b_ conversion formulas from SFT_SIRI_ and SFT_BROZEK_ and the population-specific equation from SFT_G-A_ would not be accurate when applied to a group of males and female with DS. These findings indicate that future research should be conducted to determine the external validity of the new SFT_NICKERSON_ body fat equation when used in children and adults with DS.

Research on the validation of SFT in individuals with DS is extremely limited. Accordingly, the current study uniquely adds to a gap in the literature. For instance, SFT_G-A_ was not cross-validated when initially developed by researchers due to a small overall study sample of males (*n* = 16) and females (*n* =7) with DS [28]. As a result, this is the first known study to externally validate SFT_G-A_ in people with DS. The inability to cross-validate SFT_G-A_ when initially developed is similar to issues with the current study and the newly developed SFT_NICKERSON_ equation. For instance, the small sample size of the current study did not allow the cross-validation of the newly developed equation (SFT_NICKERSON_) in a separate cohort. Altogether, obtaining a sufficient sample size that allows for development and cross-validation samples is often difficult when working with special populations.

One advantage of SFT_G-A_ is the need for only a triceps measurement. This is appealing due to its ease and convenience. For instance, our team noticed many subjects with DS appeared to experience more discomfort with skinfold calipers, commonly known as the “pinch test”, than other body composition methods. Thus, a quick skinfold assessment that minimizes measurements and time likely reduces burden experienced during testing. Our team highly recommends being considerate of participant comfort when seeking to develop any new body composition tests in people with DS. Accordingly, SFT_NICKERSON_, which only requires two skinfold measures of the mid-axilla and suprailium, is a quick and efficient body composition test for this special population.

Current study findings are similar to prior research that evaluated anthropometric-based body fat methods in individuals with DS [34,35,36]. For instance, Nickerson et al. [34] found that the body adiposity index produced significant proportional bias when compared to DXA in people with DS. Further, the body adiposity index could overestimate and underestimate %Fat as much as 17.52 and 12.21%, respectively, when applied in people with DS [34]. Similarly, Esco et al. [35] revealed BMI-based body fat equations produced large 95% LOAs (±9.77 to 17.00%) and negative proportional bias when employed in individuals with DS. Although SFT_G-A_ was developed in children with DS, it does not appear to add any more precision than previously developed anthropometric-based methods when employed in children and adults with DS.

Ideally, the current study would have obtained an adequate sample size to stratify by age (children and adults). However, it is worth noting that 70% of our sample consisted of adults. Thus, we retrospectively analyzed data of adults with DS (*n* = 14) and found that results were similar to that of the entire sample (*n* = 20) when evaluating %Fat (e.g., SEEs 3.54 to 7.65%; *r*-values 0.75 to 0.87). These findings suggest that the inclusion of children and adults may have had a minimal impact on testing outcomes. Furthermore, it does not change the main take-home message, which was the large amount of error observed when using SFT_SIRI_, SFT_BROZEK_, and SFT_G-A_ in children and adults with DS.

The reasons for the discrepancies are likely multi-factorial. Unlike SFT_G-A_, the current study consisted of both children (*n* = 6) and adults (*n* = 14) with DS, as previously noted. Accordingly, future research might seek to recruit larger samples of children and adults to determine whether the accuracy of SFT_G-A_ is influenced by age. Another issue may be the use of air displacement plethysmography (ADP) as the reference method for the development of SFT_G-A_. For example, the criterion %Fat for SFT_G-A_ was based upon Siri’s pediatric equation. This is problematic since D_b_ formulas employ outlined assumptions of the density of fat-free mass (D_FFM_). Prior research has shown that D_FFM_ varies across different race/ethnicities [37,38,39,40,41], which would influence %Fat estimates that are based upon D_b_ such as ADP and SFT. However, research has yet to explore the D_FFM_ of individuals with DS. Nonetheless, it seems logical the D_FFM_ varies from the assumed constant of Siri, when testing individuals with DS, given the large CE values observed when estimating %Fat via SFT_SIRI_. As a result, future research needs to explore this area. Findings will help uncover some of the potential reasons for discrepancies when using D_b_-based methods in individuals with DS.

Other discrepancies are worth further consideration. For instance, the data collection for SFT_G-A_ took place in Spain. Moreover, the race/ethnicity of subjects were not listed for SFT_G-A_ [28]. Nonetheless, it is very likely that subjects of SFT_G-A_ were of Hispanic descent. It is worth pointing out that the present study consisted of non-Hispanic whites (*n* = 17) and non-Hispanic blacks (*n* = 3) with DS. As previously noted, the D_FFM_ varies across different races/ethnicities [37,38,39,40,41]. As a result, this may be another reason for the large error when using SFT_G-A_ in non-Hispanics with DS. In addition, the D_FFM_ for SFT_SIRI_ and SFT_BROZEK_ are assumed to be constant (1.100 g/cm^3^) [25,26]. The significantly lower CE values observed when using SFT_SIRI_ and SFT_BROZEK_ suggest that the D_FFM_ is drastically lower than the assumed constant (1.100 g/cm^3^). Accordingly, future research, employing a multi-compartment model, should seek to explore the reasons behind the large CE values observed in the current study.

The development of SFT_NICKERSON_ and SFT_G-A_ differs from SFT_SIRI_ and SFT_BROZEK_. For instance, SFT_SIRI_ and SFT_BROZEK_ are both D_b_ conversion formulas, as previously noted, which have outlined assumptions of D_FFM_. In contrast, SFT_NICKERSON_ and SFT_G-A_ both predict %Fat and avoid the need to convert D_b_ to %Fat. Eliminating this step appears to produce less group error, as indicated by the CEs, when using SFT_G-A_ over SFT_SIRI_ and SFT_BROZEK_. However, it does not appear to be better for individual estimates as indicated by the higher SEEs and 95% LOAs observed when evaluating SFT_G-A_. One possible explanation for this is the fact that SFT_G-A_ simply uses a one-site measurement (triceps) instead of a seven-site measurement. The current study data, as highlighted in Table 2, show that the triceps has the second lowest correlation coefficient of all seven-site measurements when compared to DXA-derived %Fat. Utilizing one-site might introduce error if body fat is unequally distributed across sexes. Notably, a simpler three-site skinfold measurement excludes the triceps measurement in males but includes it for females in the general population [30,42]. Thus, this may also be a factor that resulted in large errors when using SFT_G-A_.

Although the present study has many strengths, it is not without limitations. First, the current study consisted primarily of non-Hispanic whites with DS (*n* = 17). Limited research exists exploring body composition across race/ethnicity in individuals with DS. Thus, future research should seek to explore this area. Secondly, the use of DXA as a reference method could be considered problematic. As previously mentioned, SFT results for the current study suggest that the D_FFM_ of individuals with DS is significantly lower than the assumed constants employed in stand-alone body composition models. Thus, future research should seek to integrate a criterion multi-compartment model in this special population. Thirdly, the current study developed a new equation (SFT_NICKERSON_). However, we were unable to cross-validate it due to the small sample size. Accordingly, the small sample size may also be viewed as a limitation. This is an area that needs addressing in future research when working with a special population such as DS. For instance, the development of a robust equation may be challenging due to limited statistical power. Therefore, researchers might seek to establish collaborations in an effort to create multi-site testing centers designed to evaluate the body composition of people with DS. These efforts may help improve sample size and statistical power, which may result in a more accurate body composition prediction method.

## 5. Conclusions

The current study uniquely demonstrated that a SFT equation developed in children with DS (SFT_G-A_) does not add any benefit over currently existing D_b_ conversion formulas (SFT_SIRI_ and SFT_BROZEK_). Practitioners should employ caution prior to using any of the currently evaluated SFT methods. For example, the significant CE values suggest that %Fat is grossly underestimated in people with DS, which is likely due to lower D_FFM_ values than the assumed constant previously observed in cadavers. This might erroneously suggest that individuals with excessive adiposity possess values that are normal and in a healthy range. Nonetheless, the current study developed a new equation (SFT_NICKERSON_) that can easily be administered in people with DS in a quick and efficient time frame. Nonetheless, future research should seek to determine the accuracy of the new equation in people with DS. Furthermore, future research needs to explore differences in body composition across race/ethnicity for people with DS.

## Figures and Tables

**Table 1:** Subject characteristics (mean and standard deviation (SD) and range).

	Mean	SD	Minimum	Maximum
Height (cm)	148.49	8.77	134.62	162.56
Weight (kg)	64.65	18.00	37.64	101.00
Age (yrs.)	22	10	10	43
BMI (kg/m^2^)	43.38	11.10	25.76	62.62
SS (mm)	154.28	60.87	45.00	273.20
SFT_G-A_	29.55	12.07	13.13	52.46
SFT_SIRI_	24.24	10.26	4.34	42.56
SFT_BROZEK_	23.63	9.47	5.26	40.55
DXA (%Fat)	37.14	13.22	8.90	56.40

BMI = body mass index; SS = sum of seven-site skinfolds; SFT = skinfold thickness; DXA = dual energy X-ray absorptiometry.

**Table 2:** Agreement between SFT methods and DXA in individuals with DS (*n* = 20).

					Pearson’s Correlation		Bland-Altman Analysis			
Method	(Mean ± SD)	*p*-Value	ES	SEE	r	*p*-Value	CE ± 1.96 SD	Trend	Upper	Lower
SFT_G-A_	29.55 ± 12.07	<0.001	0.60	8.60	0.72	<0.001	−7.59 ± 18.65	−0.13	11.06	−26.23
SFT_SIRI_	24.24 ± 10.26	<0.001	1.09	3.75	0.93	<0.001	−12.90 ± 10.10	−0.58	−2.80	−23.01
SFT_BROZEK_	23.63 ± 9.47	<0.001	1.17	3.47	0.93	<0.001	−13.51 ± 10.82	−0.69	−2.69	−24.33
DXA	37.14 ± 13.22	---	---	---	---	---	---	---	---	---

SFT = skinfold thickness; DXA = dual energy X-ray absorptiometry; ES = effect size; SEE = standard error of estimate; CE = constant error; SD = standard deviation.

**Table 3:** Correlation between skinfold sites and DXA-derived %Fat.

	*r*	*p*-Value
Chest	0.52	0.019
Triceps	0.67	0.001
Subscapular	0.73	<0.001
Mid-axilla	0.82	<0.001
Abdomen	0.79	<0.001
Suprailium	0.79	<0.001
Thigh	0.75	<0.001

**Table 4:** Contribution and order of entry of prediction variables to SFT_NICKERSON_.

Model and Variables	Equation	*r*	R^2^	SEE	*p*-Value
Mid-Axilla	%Fat = 17.747 + (0.982 × mid-axilla)	0.81	0.66	7.94	<0.001
Mid-Axilla + Suprailium	%Fat = 10.323 + (0.661 × mid-axilla) + (0.712 × suprailium)	0.91	0.83	5.76	<0.001

## Data Availability

The data used in this study is not available for public sharing or distribution due to confidentiality and privacy concerns.
